# Extracellular vesicles in renal cell carcinoma: challenges and opportunities coexist

**DOI:** 10.3389/fimmu.2023.1212101

**Published:** 2023-07-04

**Authors:** Yukang Lu, Mengting Zhang, Jiajun Zhou, Xiulan Liu, Lanfeng Wang, Xinyi Hu, Yiping Mao, Rongfa Gan, Zhiping Chen

**Affiliations:** ^1^ The First School of Clinical Medicine, Gannan Medical University, Ganzhou, China; ^2^ Department of Laboratory Medicine, First Affiliated Hospital of Gannan Medical University, Ganzhou, China; ^3^ Department of Medical School, Kunming University of Science and Technology, Kunming, China; ^4^ Department of Nephrology, First Affiliated Hospital of Gannan Medical University, Ganzhou, China

**Keywords:** extracellular vesicles, renal cell carcinoma, tumor microenvironment, engineered extracellular vesicles, tumor vaccine

## Abstract

Renal cell carcinoma (RCC) represents an extremely challenging disease in terms of both diagnosis and treatment. It poses a significant threat to human health, with incidence rates increasing at a yearly rate of roughly 2%. Extracellular vesicles (EVs) are lipid-based bilayer structures of membranes that are essential for intercellular interaction and have been linked to the advancement of RCC. This review provides an overview of recent studies on the role of EVs in RCC progression, including involvement in the interaction of tumor cells with M2 macrophages, mediating the generation of immune tolerance, and assuming the role of communication messengers in the tumor microenvironment leading to disease progression. Finally, the “ troika “ of EVs in RCC therapy is presented, including engineered sEVs’ or EVs tumor vaccines, mesenchymal stem cell EVs therapy, and reduction of tumor-derived EVs secretion. In this context, we highlight the limitations and challenges of EV-based research and the prospects for future developments in this field. Overall, this review provides a comprehensive summary of the role of EVs in RCC and their potential as a viable pathway for the future treatment of this complex disease.

## 1 Introduction

Renal Cell Carcinoma (RCC) is among the most prevalent kind of kidney cancer, which accounts for approximately 90% of all occurrences. The American Cancer Society estimates that there will be roughly 76,080 cases reported and 13,780 deaths from RCC in the USA in the year 2021 (1). The prevalence of RCC has been progressively growing over the last several decades, and the level is predicted to continue to climb as a result of causes such as population aging and changes in lifestyle and environmental exposure. and it is expected to continue to rise due to factors such as population aging and changes in lifestyle and environmental exposure. Clear cell renal cell carcinomas (ccRCC) are the more frequent subtype, accounting for approximately 80-90% of all occurrences (2). In the early stage, many RCC patients have no obvious clinical symptoms and tumor markers are not sensitive to the early diagnosis of RCC, so about 16% of RCC patients already have metastasis at the time of diagnosis (3). The 5-year survival rate for early-stage RCC typically exceeds 90%, indicating a high proportion of patients survive within 5 years of treatment initiation. Surgical resection represents the most efficacious treatment modality for early-stage RCC, particularly when the disease is detected early. In contrast, the 5-year survival rate for advanced RCC is usually low, ranging between 30 and 40 percent (4). The choice of treatment and prognosis for advanced RCC is dictated by several factors, including tumor size, location, pathological type, and grade. However, the outlook for advanced RCC remained bleak, having only a 12% five-year survival rate for stage IV cancer (5). Moreover, existing therapies often cause significant side effects and are associated with high costs and risks of treatment resistance and disease relapse. Therefore, research on the diagnosis, disease progression, and treatment of RCC is crucial to save patients’ lives and improve their quality of life.

Extracellular vesicles (EVs) are small, membrane-encased nanoparticles that are purposely released into the extracellular milieu by cells (6). Initially, EV secretion is found in reticulocytes and is thought to be the mechanism for the removal of excess membrane proteins, therefore, corresponding studies were scarce; nonetheless, the varied and significant potential for EVs was brought to the fore when sEVs were discovered to carry potent biological molecules and play critical functions in communication between cells (7). According to MISEV2018, EVs have broadly classified into two types according to their sizes: sizes < 200 nm are called small extracellular vesicles (sEVs), and sizes > 200 nm are large extracellular vesicles (lEVs) (8). This review will be named using sEVs or LEVs according to the extracellular vesicle size of the original authors, and EVs will be used uniformly for cases where the size of the vesicles is not specified in the original text, or when some concepts are being described. EVs include a diverse range of bioactive compounds, such as proteins, nucleic acids, and lipids, which may be ingested by various cells and bestow a variety of regulatory effects. These particles are recognized as essential mediators of interaction between cells and continue to be a focus of study into their potential utility in diagnostic and therapeutic contexts for a variety of disorders, including cancer (9).

In RCC, EVs have been shown to have a vital function in tumor growth and metastases through their capacity to stimulate angiogenesis, evade detection by the immune system, and bolster drug resistance mechanisms (10). The accessibility of EVs from a multiplexity of biological fluids positions them as an appealing avenue for the non-invasive detection and monitoring of RCC (11). And because EVs, as lipid bilayer vesicles, can effectively protect their internal RNA from hydrolysis, their use as anti-cancer drug carriers, tumor vaccines, and therapeutic targets has received increasing attention from a wide range of researchers (12).

## 2 EVs in RCC deterioration

**Figure 1:**
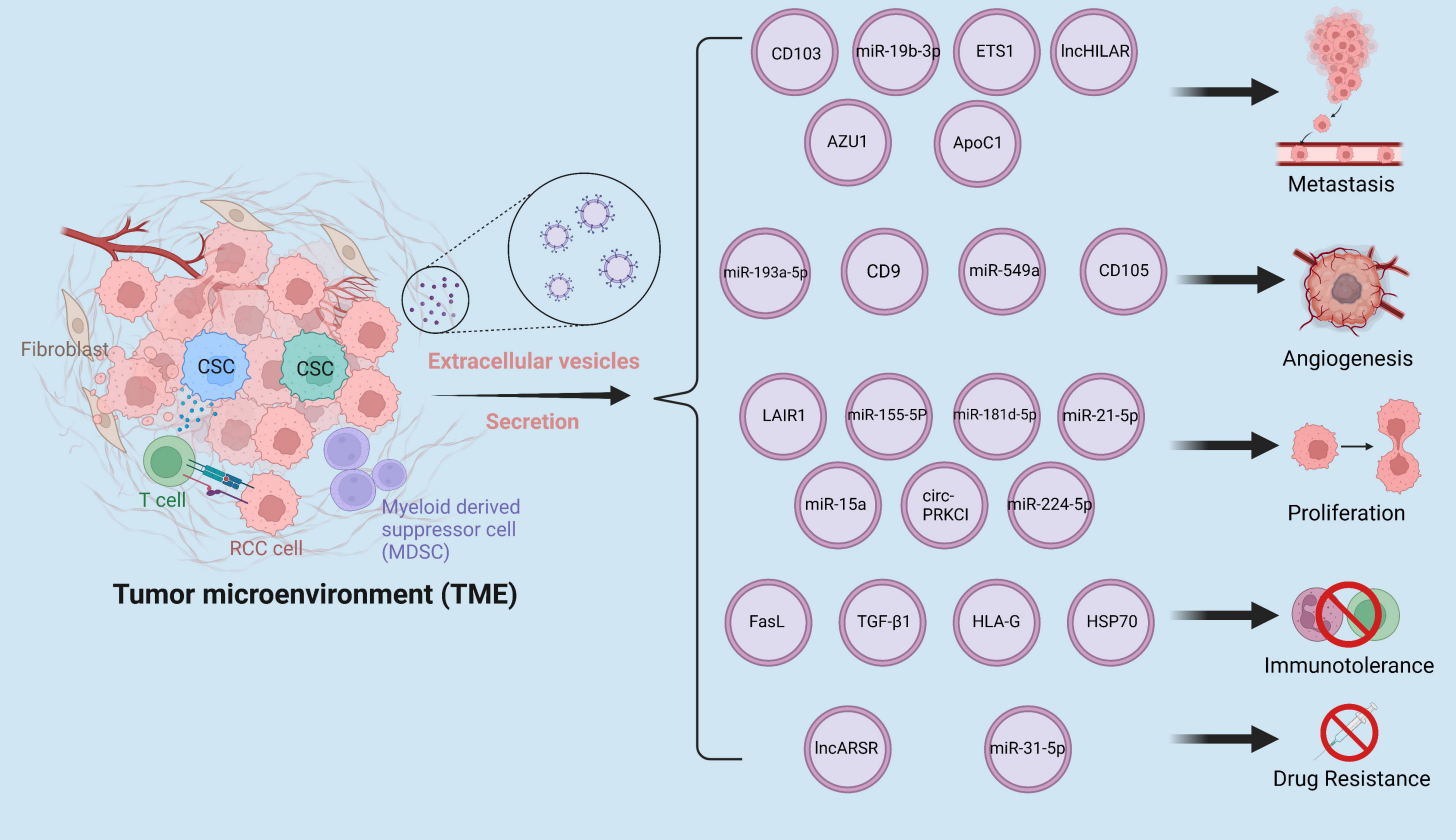
The tumor microenvironment is a complex milieu composed of various components that interact with one another through the secretion of EVs. These EVs facilitate the dissemination of pro-cancer cargoes, leading to enhanced metastasis, angiogenesis, proliferation, drug resistance, and immune evasion in RCC. The interplay of these components within the tumor microenvironment has been implicated in the progression and aggressiveness of RCC, highlighting the critical role of EVs in modulating tumor behavior.

The tumor microenvironment is a complex environment made up of many kinds of cells, including stromal cells, fibroblasts, vascular endothelial cells, immune cells, and the extracellular matrix, all of which play an important role in tumor genesis and development (13). EVs, which are high in proteins, lncRNAs, and microRNAs, enable communication between cells in the tumor microenvironment and these EVs indirectly or directly contribute to the deterioration of RCC (**Figure 1**).

### 2.1 RCC-Derived EVs in the immune system

#### 2.1.1 EVs in the vicious circle of M2 macrophages and RCC cells

Tumor-associated macrophage (TAM) are macrophages that infiltrate tumor tissues and are derived from circulating peripheral blood mononuclear cells (14). Tumor cells secrete a plethora of chemokines, including colony-stimulating factor-1 and interleukins, to attract monocytes from the peripheral blood circulation into the tumor microenvironment (TME), ultimately inducing monocytes to differentiate into macrophages (15). Broadly speaking, TAMs primarily consist of classically activated macrophages (M1) and alternatively activated macrophages (M2), with M1 macrophages exhibiting anti-tumor actions and M2 macrophages supporting tumor development, invasion, as well as metastasis (16). Macrophages infiltrating in the periphery of malignant tumor tissues are mostly of M2 type, so many places define M2 form macrophages as TAM in a narrow sense. M0 macrophages are in an unactivated, immature state. M0 macrophages have the function of phagocytosis and removal of cellular debris, as well as the initiation of the inflammatory response. m1 and m2 macrophages have different roles in the immune response and inflammatory process, whereas m0 macrophages are in a neutral state and have undifferentiated characteristics. The switch from the M0/1 to M2 phenotype of macrophages may contribute to cancer development by boosting cell proliferation, metastasis, drug dependency, and immune evasion (17). Recent studies have shed light on the role of EVs in mediating this communication. Specifically, EVs secreted by RCC cells have been shown to promote a malignant cycle that drives tumorigenesis and metastasis. These EVs are taken up by TAMs, leading to changes in gene expression and altered cellular behavior. The next sections will mostly investigate the close interaction between M2 macrophages and RCC cells.

Huang et al. (18) reported that sEVs secreted by RCC cells contain markedly elevated levels of circSAFB2. These sEVs were observed to be internalized by macrophages, where they targeted miR-620 in spongy macrophages. This led to a significant increase in JAK1 and STAT3 protein expression, ultimately resulting in the activation of the JAK1-STAT3 pathway. Consequently, M2 polarization was promoted in macrophages. Importantly, the authors demonstrated through both *in vivo* and *in vitro* experiments that macrophages with elevated expression of circSAFB2 can, in turn, stimulate the proliferation, invasion, and *in vivo* metastatic ability of RCC cells. These findings provide novel insights into the intricate interplay between sEVs, macrophages, and RCC cells, highlighting the potential of circSAFB2 as a viable therapeutic target in the management of RCC. Moreover, RCC-derived sEVs exhibited high levels of long non-coding RNAs (lncRNAs) with pro-cancer effects, including lncARSR and AP000439.2. Notably, macrophages that received lncARSR delivered by tumor cells demonstrated a significant increase in CD163 and CD206 expression, along with a rise in anti-inflammatory cytokines TGFβ-1 and IL-10 secreted by macrophages. Conversely, the levels of pro-inflammatory cytokines IL-6, IL-12, and IL-1β decreased, indicating that macrophages underwent M2 polarization, a process thought to be mediated through the activation of the STAT3 signaling pathway (19). However, this study did not reveal the specific mechanism by which lncARSR exerts its role in promoting M2 polarization. In a separate study on lncARSR-mediated sunitinib resistance in RCC, lncARSR was found to act through the competitive binding of miR-34 and miR-449 (20). Nevertheless, the downstream molecular mechanisms of lncARSR during the occurrence of M2 polarization in macrophages require further research. Likewise, AP000439.2 was observed to induce M2 polarization by stimulating STAT3 phosphorylation in macrophages, activating the NF-κB signaling pathway, and subsequently triggering M2 polarization in macrophages upon exposure to AP000439.2. Ultimately, this process accelerated ccRCC migration (21).

The aforementioned studies have underscored the significance of RCC-derived EVs in driving M2 polarization of macrophages within the tumor microenvironment. We will now discuss several investigations that have explored the ability of M2 macrophages, activated by EVs, to promote RCC progression. In the hypoxic microenvironment, TAMs demonstrate a significant enrichment of miR-155-5P, which may be transmitted to RCC cells via sEVs. miR-155-5P plays a crucial role in promoting the activation of the PI3K/AKT signaling cascade by enhancing the mRNA stability of *insulin-like growth factor 1 receptor* (*IGF1R*) through the human antigen R (HuR) pathway. Ultimately, this contributes to the malignant transformation of RCC cells. Remarkably, this represents a rare example of a miRNA exerting a positive regulatory effect on its target gene (22). In a study by Zhang et al. (23), M2 macrophages were found to transmit miR-21-5p to RCC cells through sEVs. This miRNA targets the 3’ untranslated regions (3’UTRs) of *PTEN*, an oncogene encoding PIP3 phosphatase, which dephosphorylates PIP3 to PIP2, thereby exerting negative regulation over the PI3K/Akt signaling pathway. Upon *PTEN* downregulation, Akt signaling is overactivated, ultimately promoting the proliferation, invasion, and metastasis of RCC cells. Liu et al. (24) revealed that hypoxia-inducible factor 1A (HIF1A) was markedly upregulated in TAMs. HIF1A binds to hypoxia-response element 2 (HRE2) in the miR-193a promoter region, thereby initiating the transcription of miR-193a-5p in TAMs. Subsequently, the upregulated miR-193a-5p is sorted into sEVs by TAMs. Upon uptake by tumor cells, the highly expressed miR-193a-5p promotes tumor angiogenesis, invasion, and proliferation by sponge-adsorbing tissue inhibitor of metalloproteinases 2 (TIMP2).

**Figure 2:**
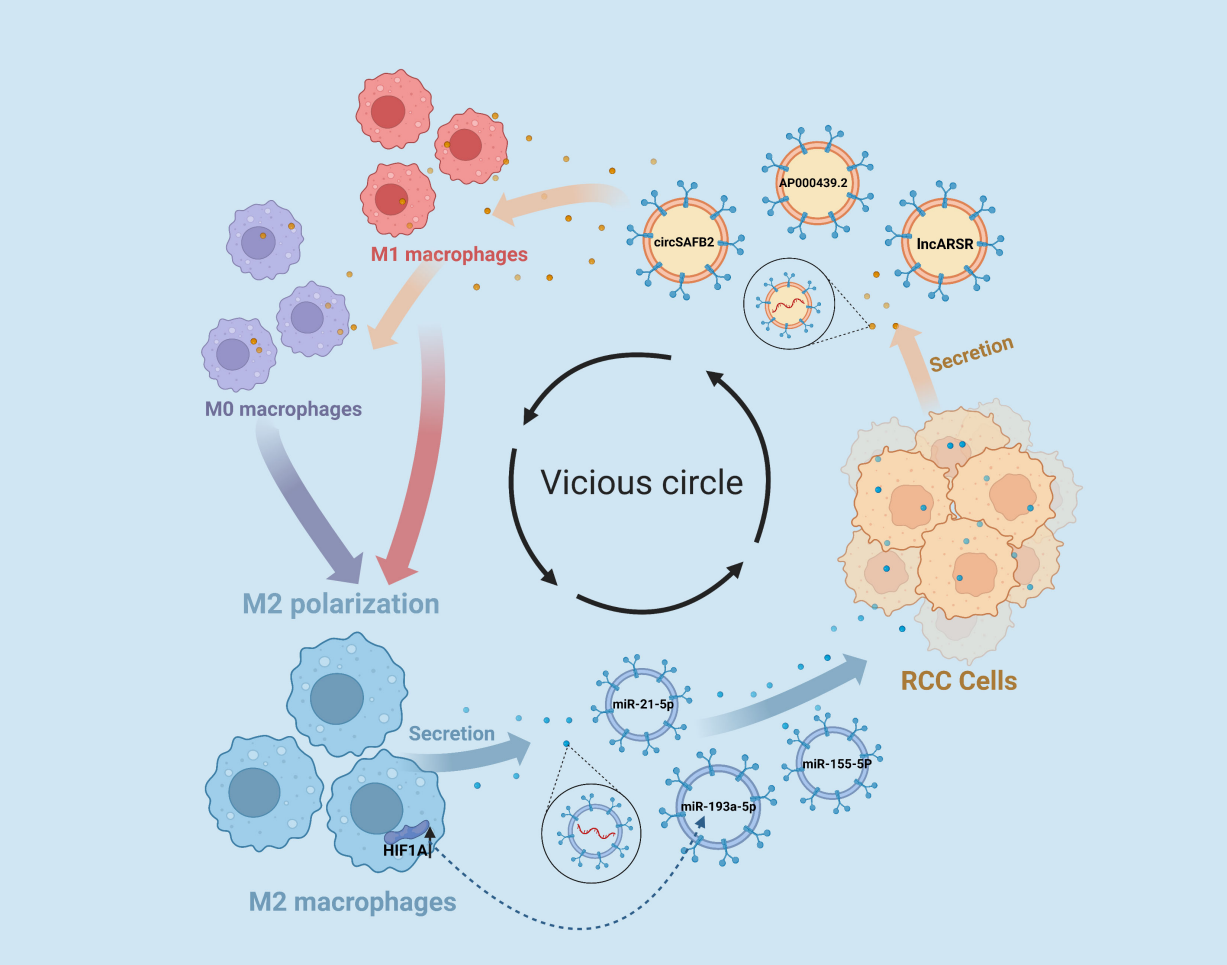
EVs Contribute to the Vicious Cycle of RCC-M2 Macrophage Interactions. RCC cells release extracellular vesicle EVs enriched with the lncARSR and AP000439.2, as well as the circular RNA circSAFB2, which play a significant role in promoting M2 macrophage polarization within the tumor microenvironment. Furthermore, M2 macrophages can reciprocate by releasing EVs containing microRNAs miR-193a-5p, miR-21-5p, and miR-155-5p that promote malignant transformations in RCC cells.

The intricate crosstalk between RCC cells and TAMs, mediated by EVs, creates a vicious cycle that fuels tumorigenesis and fosters malignant transformations within the tumor microenvironment. RCC cells secrete EVs that activate TAMs, which in turn secrete EVs containing oncogenic factors that promote the proliferation, invasion, and metastasis of RCC cells. This reciprocal communication between RCC cells and TAMs not only exacerbates the malignant phenotype of RCC but also contributes to the formation of an immunosuppressive tumor microenvironment, thereby facilitating tumor immune evasion and therapy resistance (**Figure 2**).

#### 2.1.2 Involvement in immune escape

Tumor cells can present tumor-specific antigens, which are recognized by various immune cells, allowing the immune system of the body to recognize and remove cancerous cells. Tumor cells, on the other hand, can modify their surface antigens to avoid immune detection, which enables tumor progression and metastasis (25). In addition, recent studies have shown that tumor-derived EVs can exert immunosuppressive effects by inhibiting the immune system, thereby promoting tumor immune evasion and facilitating tumor progression (26). These findings highlight the critical role of tumor-derived EVs in shaping the tumor microenvironment and regulating the immune response and suggest that targeting EVs-mediated immunosuppression may represent a promising therapeutic strategy for cancer treatment.

Wang et al. have demonstrated that tumor cell-derived sEVs are pivotal in driving immune escape and drug resistance in ccRCC via a complex interplay of signaling pathways, including mTOR, ERK, STAT, and NF-κB. However, the specific cargo molecules within these sEVs that are responsible for this biological effect remain unknown (27). It was found that RCC cell-derived sEVs can significantly suppress the secretion of cytokines, such as interleukin-2 and interferon-gamma, by T cells, and in addition, the presence of Fas ligands within these sEVs can induce T cell apoptosis through binding with Fas, thereby promoting immune evasion by tumor cells (28). Despite the crucial role of NK cells in tumor immunity, their anti-tumor activity can be impeded by impaired degranulation and cytokine secretion in TGF-β1-enriched tumor microenvironments, which Xia et al. (29) demonstrated can be induced by TGF-β1 transported by RCC cell-derived sEVs through the phosphorylation of Smad2 and Smad3, resulting in defective NK cell function. CD105^+^ renal cancer stems cells-derived sEVs serve as crucial communication molecules that impede the differentiation and maturation of monocytes into dendritic cells (DCs). This inhibition of DC differentiation has been shown to suppress the proliferation of CD3^+^ T lymphocytes in the TME, resulting in immunological escape. The identification of human leukocyte antigen-G (HLA-G) as a key factor in this process suggests that targeted therapies aimed at disrupting the sEV-mediated delivery of HLA-G may hold promise for enhancing the effectiveness of anti-tumor immune responses in the context of RCC (30). Further studies are needed to elucidate the precise mechanisms underlying the immunosuppressive effects of sEVs and to identify additional targets for therapeutic intervention. Gao (31) and Diao (32) independently reported that RCC cell-derived sEVs had high expression of heat shock protein 70 (HSP70), which is important in the formation of immunological tolerance. In their studies, HSP70 was found to act through two distinct mechanisms. The first mechanism involves initiating antigen-specific immunosuppression of myeloid-derived suppressor cells (MDSCs) in a toll-like receptor 2 (TLR2)-dependent manner, leading to antigen-specific immunosuppression of cytotoxic T lymphocytes (CTLs). The second mechanism involves stimulating the STAT3 signaling pathway to promote MDSCs expression, eventually culminating in immune escape. These findings highlight the critical role of RCC cell-derived sEVs in modulating the immune microenvironment and suggest that HSP70 may be a promising therapeutic target for the development of novel RCC immunotherapies.

### 2.2 Mediating epithelial-mesenchymal transition

The epithelial-mesenchymal transition (EMT) is a crucial phase in cancer cell metastasis in which epithelial traits are lost and mesenchymal qualities are acquired, ultimately conferring stronger invasive capabilities, stem-cell properties, immunosuppressive abilities, and drug resistance upon cancer cells (33). Several studies have established that the dissemination of cancer-derived EVs is critical in inducing EMT (34).

Wang et al. (35) found that RCC stem cells secrete more CD103^+^ sEVs, and these CD103^+^ sEVs fulfill a “pathfinder” role, leading RCC cells to diverse metastatic sites. Notably, CD103^+^ deficiency severely impacts the RCC cells’ capacity to colonize distant organs. Concurrently, these sEVs serve as carriers of miR-19b-3p to target recipient cells and orchestrate EMT in ccRCC by targeting PTEN, thus heightening RCC cells’ metastatic capacity. Li et al. (36) observed an abnormal increase in the level of apolipoprotein C1 (ApoC1) in ccRCC, with survival rate negatively correlated to ApoC1 levels. They further showed that ApoC1 induces EMT in ccRCC via massively stimulating STAT3 activation. Importantly, in this process, sEVs carry ApoC1 from ccRCC cells to vascular endothelial cells and induce EMT via STAT3 activation, consequently boosting the proliferative, invasive, and metastatic abilities of ccRCC cells. Additionally, Jin et al. (37) discovered that sEVs derived from RCC cells transmit metastasis-associated lung adenocarcinoma transcript 1 (MALAT1), which orchestrates TFCP2L1 expression by modulating the transcription factor ETS1, ultimately promoting EMT in RCC cells.

In conclusion, we have summarized the important role of EVs in regulating EMT in RCC. However, it is vital to acknowledge that the regulation of EMT is a multifaceted process that is intricately linked to the entire process of tumor development, involving a complex interplay of diverse factors and signaling pathways (38). Notably, despite the growing recognition of the importance of EVs in EMT induction in RCC, the number of studies exploring this phenomenon remains limited. Therefore, future research endeavors should aim to broaden our understanding of the complex interactions between EVs and other factors involved in EMT regulation, rather than limiting the focus to a single cargo of EVs. Such a holistic approach would provide a more comprehensive understanding of RCC progression and metastasis, ultimately paving the way for the development of novel therapeutic strategies to curtail RCC metastasis and improve patient outcomes.

### 2.3 Promotes proliferation, invasion, and migration

In the TME, EVs of cancer-associated fibroblasts (CAFs) play a significant part in the development of RCC. Fu et al. (39) demonstrated that sEVs released by CAFs are effectively internalized by RCC cells. These sEVs were found to enhance the invasive properties of RCC cells by upregulating proteins implicated in metastasis, including fibronectin, N-cadherin, vimentin, MMP9, and MMP2. Furthermore, these sEVs were shown to modulate cell cycle progression, enhancing the proliferative and anti-apoptotic capacities of RCC cells unfortunately, they did not specify which cargoes in sEVs mediated these functions. Ding et al. (40) found that sEVs delivered by CAFs were enriched with miR-181d-5p. These CAF-derived sEVs were effectively internalized by RCC cells, where the high levels of miR-181d-5p led to the activation of the Wnt/β-catenin signaling pathway. Specifically, miR-181d-5p was found to bind to the 3′-UTR of the tumor suppressor *RNF43*, resulting in the downregulation of its protein expression. This event ultimately resulted in enhanced cancer stemness and tumor growth. Additionally, sEVs derived from CAFs are capable of delivering miR-224-5p to ccRCC cells, thus contributing to their malignant changes (41). These findings underscore the critical role played by CAF-derived EVs in RCC pathogenesis. However, further studies are warranted to unravel the intricate mechanisms underlying the functional interplay between CAF-derived EVs and RCC cells and to identify specific cargo molecules that can serve as potential therapeutic targets.

**Figure 3:**
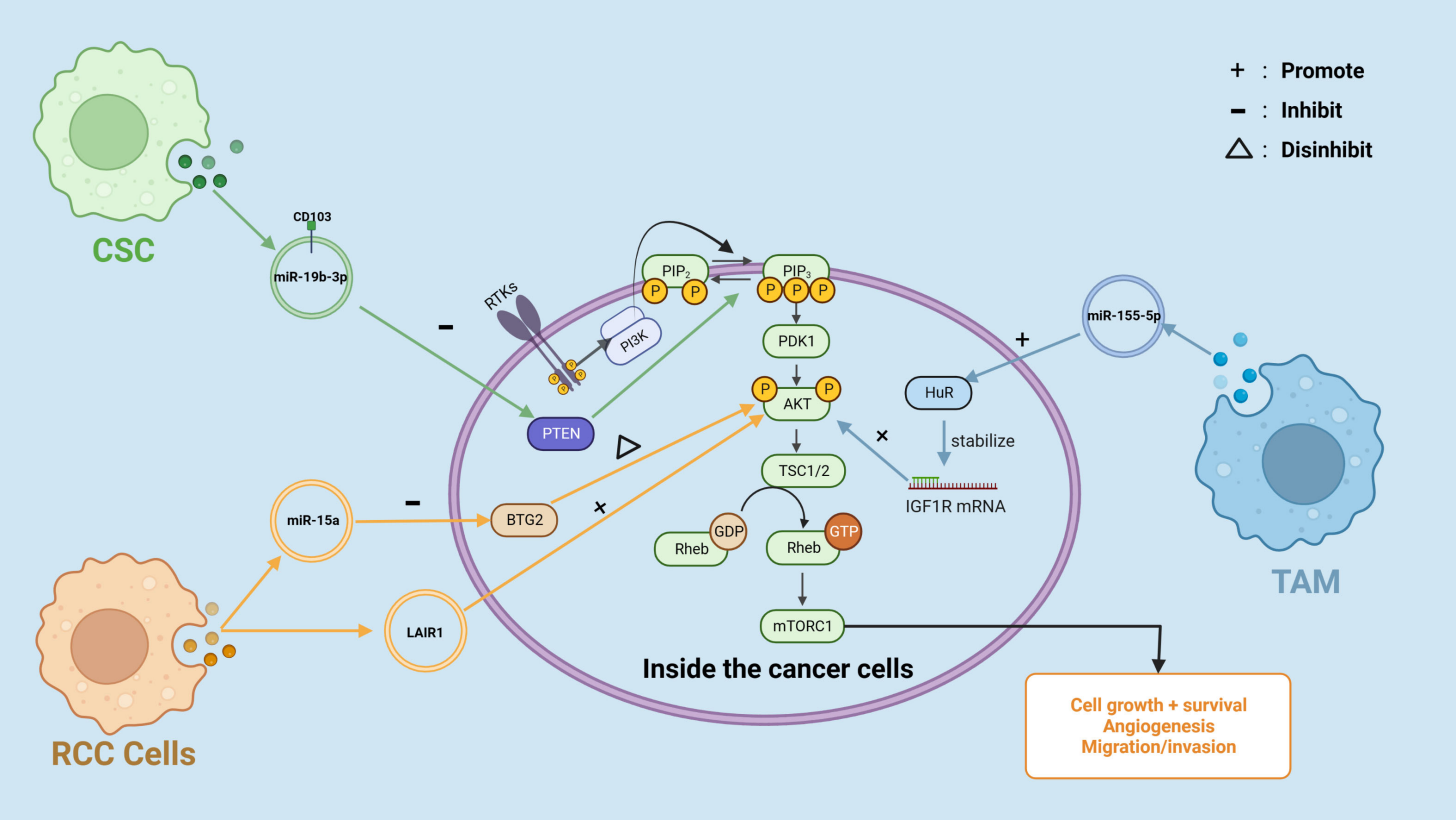
Cancer-derived EVs are involved in the PI3K/AKT pathway in RCC.

In RCC, Cancer-derived sEVs have been demonstrated to downregulate hepatocyte cell adhesion molecule (hepaCAM) expression in RCC in a p-AKT-dependent way (42). According to a previous study by Zhang et al. (43), hepaCAM was discovered to arrest RCC cells in the G1 stage of the cell cycle, resulting in cell proliferation suppression, and thus RCC-derived sEVs-mediated downregulation of hepaCAM may be associated with excessive proliferation of RCC cells (42). Moreover, highly malignant RCC cells can pass circ-PRKCI to less malignant RCC cells via sEVs, and transferred circ-PRKCI acts as a sponge for miR-545-3p, increasing the expression of Cyclin D1 (CCND1), a cell cycle regulator associated with cell proliferation in a variety of tumor cells, and overexpression of CCND1 ultimately increased proliferation, migration and invasion of RCC cells (44). Cell-secreted EVs in the tumor microenvironment have been shown to play an important role in the PI3k/AKT signaling pathway, promoting the malignant progression of RCC (**Figure 3**). For instance, Li et al. (45) discovered that sEVs generated by RCC cells can regulate the activity of RCC cells via miR-15a. B-cell translocation gene 2 (BTG2), a miR-15a downstream gene, had a negative connection with miR-15a expression. RCC cells release sEVs containing miR-15a, which stimulates the PI3K/AKT signaling pathway by inhibiting BTG2 expression, hence speeding RCC proliferation. In another insightful study conducted by Jingushi et al. (46) immunoglobulin-like receptor 1 (LAIR1) was significantly enhanced in tumor-derived EVs and capable of increasing RCC cell proliferation and development of tumors via AKT phosphorylation.

### 2.4 EVs in hypoxic microenvironments

Many solid tumors, including renal cell carcinoma (RCC), are characterized by a hypoxic microenvironment, which is a hallmark of the tumor microenvironment and is closely associated with cancer progression and poor prognosis (47). Hypoxia in the tumor microenvironment triggers a complex network of molecular pathways, such as hypoxia-inducible factor signaling, to promote tumor growth, angiogenesis, and metastasis (48). In RCC, the hypoxic microenvironment is particularly notable due to the high metabolic demands of the tumor and the limited vascularization in the surrounding tissue (49). In this context, EVs derived from hypoxic cancer cells have been implicated in promoting RCC progression and metastasis by transferring oncogenic cargoes, including miRNAs and proteins, to recipient cells. Notably, these hypoxia-induced EVs have been shown to modulate key biological processes, including angiogenesis, immune evasion, and metastasis, with significant implications for RCC pathogenesis.

As previously mentioned, in the hypoxic tumor microenvironment, TAMs have been shown to deliver miR-155-5p to RCC cells via sEVs. This miRNA has been shown to enhance the stability of *IGF1R* mRNA via the RNA-binding protein HuR, thereby promoting the activation of the PI3K/AKT signaling cascade (22). There have been additional reports concerning miR-155 and its role in response to hypoxic conditions. In RCC, sEVs secreted by hypoxic RCC cells were found to be 2.03-2.37 times higher than those secreted under normoxic conditions. Furthermore, sEVs secreted by RCC cells were found to contain higher levels of miR-155. This miRNA was shown to downregulate the expression of tumor suppressor genes *p15*, *p21*, and *Gadd45*, as well as apoptosis-related genes *Bim* and *TRAIL*, by targeting the phosphorylation of Forkhead Box O3 (FOXO3) in normoxic RCC cells (50). This mechanism inhibited cell apoptosis while promoting cell migration and proliferation. Samoylenko et al. (51) employed time-gated Raman spectroscopy (TG-RS) to study the sEVs generated by RCC cells in a hypoxic tumor microenvironment. This innovative technique enabled the authors to achieve improved signal-to-noise ratios, allowing for a more robust analysis of the sEVs ‘ protein content. They reported a significant upregulation of cell adhesion and CD9 proteins in these sEVs, suggesting their potential involvement in RCC tumor progression. These findings highlight the utility of advanced spectroscopic techniques in characterizing the complex molecular landscape of sEVs and their role in modulating RCC pathogenesis and can be applied to other cancers as well. RCC cells residing in hypoxic microenvironments secrete sEVs containing hypoxia-induced LncRNA associated with RCC (lncHILAR). Upregulation of lncHILAR in RCC cells is largely attributed to aberrant methylation and acetylation following hypoxic exposure. The uptake of these sEVs by normoxic RCC cells leads to the upregulation of their target gene CXCR4. This process occurs not only by sponging miR-613/206/1-1-3p but also indirectly via the activation of the target Jagged1/Notch pathway. The elevated CXCR4 levels ultimately promote the metastatic capacity of RCC cells (52). Moreover, in response to the hypoxia-induced by rapid proliferation, hypoxic RCC cells were found to secrete increased levels of small extracellular vesicles (sEVs) containing carbonic anhydrase 9 (CA9) (53). CA9 is known for its high expression in the hypoxic microenvironment of many solid tumors and is believed to play a crucial role in promoting angiogenesis. In RCC, these sEVs are thought to facilitate angiogenesis by transporting CA9 in response to nutrient deprivation in the hypoxic microenvironment (54). In summary, EVs hold immense potential as therapeutic targets for RCC, especially in the context of the hypoxic tumor microenvironment. Further investigations are warranted to elucidate the precise mechanisms underlying the functional interplay between hypoxia-induced EVs and recipient cells, with a focus on identifying specific cargo molecules that can be targeted to curtail RCC progression and improve patient outcomes.

### 2.5 Pro-angiogenic effects or altered vascular permeability in tumor development

The formidable proliferative capacity of tumor cells necessitates an extensive amount of oxygen and nutrients which can initially rely on diffusion when the tumor volume is less than 2 mm^3^. However, as the tumor size increases, a hypoxic microenvironment will form, and the tumor must produce new blood vessels to obtain sufficient oxygen (55). This angiogenesis process optimizes the supply of nutrients to support tumor growth, while the accompanying altered vascular permeability establishes a pre-metastatic ecological niche, thus promoting cancer’s metastatic spread (56).

Lindoso et al. (57) revealed that mesenchymal stem cells (MSCs), upon uptake of EVs derived from RCC stem cells, exhibited expression elevated of MMP1, MMP3, and CXCR4, promoting angiogenesis and facilitating tumor growth *in vivo*. Nevertheless, the precise substance responsible for this effect remains unknown. Subsequently, sEVs secreted from tyrosine kinase inhibitor (TKI) -resistant RCC cells were found to have a low expression of miR-549a, leading to elevated expression of hypoxia-inducible factor-1α (HIF1α) in vascular endothelial cells. The resultant increase in tumor angiogenesis and enhanced vascular permeability contributes to the creation of a pre-transfer ecological position, facilitating tumor cells to initiate metastasis post-acquiring TKI resistance in ccRCC (58). CD105^+^ sEVs secreted by RCC stem cells contain numerous pro-angiogenic factors including VEGF, FGF2, ephrin A3, and angiopoietin 1, as well as mRNAs associated with stimulating angiogenesis like MMP2 and MMP9. CD105^+^ sEVs have a pivotal role in promoting tumor angiogenesis and establishing pre-metastatic ecological niches by delivering these mRNAs (59). Earlier, Jingushi et al. (60) extracted EVs from RCC tissue samples and compared them to EVs from adjacent normal kidney tissue. They found that tumor tissue-derived sEVs were rich in azurocidin 1 (AZU1) and effectively altered vascular endothelial cell layer permeability. In a follow-up study (61), it was established that AZU1 is packaged into sEVs via N-linked glycosylation, which alters vascular endothelial cell layer permeability by elevating intracellular Ca2^+^ concentrations. These findings suggest that AZU1 glycosylation inhibition could be a promising RCC treatment strategy.

### 2.6 Induces development of therapeutic resistance

Tumor drug resistance has been a longstanding challenge in the treatment of various malignancies. With the increasing focus on EVs, increasing data shows that they exert a significant part in transferring drug resistance among tumor cells (62). Advanced RCC, in particular, is highly vulnerable to drug resistance, which significantly impacts patient survival quality. Around twenty percent of terminal RCC patients are unresponsive to TKI treatment (e.g., sunitinib and sorafenib), while patients who display early treatment response often develop drug resistance, resulting in tumor development after 15 months of treatment (63).

The propagation of drug resistance between resistant and sensitive cells is a complex process governed by intercellular communication mechanisms, with EVs playing a critical role. These EVs serve as vehicles for the transfer of bioactive molecules, including proteins, lipids, and nucleic acids, between cells in the tumor microenvironment, facilitating the spread of drug resistance. In our previous discussion, we highlighted the ability of lncARSR to mediate the M2 polarization of macrophages via sEVs in RCC. In this section, we will discuss the research conducted by Qu et al. on the association between lncARSR and sunitinib resistance in RCC. Qu et al. (20) observed that activated AKT in sunitinib-resistant RCC cells downregulates FOXO1 and FOXO3a, which subsequently activates the transcription of lncARSR, resulting in its high expression in drug-resistant cells. The 5’ end of lncARSR is sorted into sEVs by binding to heterogeneous nuclear ribonucleoprotein A2B1 (hnRNPA2B1). Upon uptake by sunitinib-sensitive RCC cells, these sEVs upregulate the levels of lncARSR, which acts as a sponge for miR-34 and miR-449 in target cells, thereby blocking their biological functions. Qu et al. found that miR-34 and miR-449 can target and inhibit AXL and c-MET. Consequently, the upregulation of lncARSR ultimately upregulates AXL and c-MET in sunitinib-sensitive cells, leading to the development of sunitinib resistance. Furthermore, the overactivation of AXL and c-MET is known to activate the STAT3, AKT, and ERK signaling pathways, which are also activated by the AKT signaling pathway that upregulates lncARSR in drug-resistant RCC cells. This creates a positive feedback loop, resulting in continuous transcription of lncARSR and its sorting into sEVs, leading to the widespread propagation of sunitinib resistance. Moreover, it has been observed that RCC cells treated with sunitinib and axitinib secrete a higher number of sEVs, including both large and small vesicles. In contrast, RCC cells treated with tyrosine kinase inhibitors display high levels of glucose transporter 1 (GLUT1) sorted into sEVs, which leads to increased uptake of glucose and enhanced glycolytic activity (64). This phenomenon may contribute to the development of drug resistance mechanisms in RCC. In a recent study, He et al. (65) discovered that sorafenib-resistant RCC cells secrete sEVs that contain high levels of miR-31-5p. They further demonstrated *in vitro* and *in vivo* that miR-31-5p plays a pivotal role in inducing sorafenib resistance. Upon uptake by target cells, the overexpression of miR-31-5p within the EVs promotes drug resistance by targeting the expression of MutL homolog 1 (MLH1).

Understanding the molecular mechanisms underlying EVs-mediated drug resistance and metabolic reprogramming is crucial for developing effective therapies for RCC. Targeting EVs and their cargo molecules, including lncARSR and miR-31-5p, may represent promising therapeutic strategies to overcome drug resistance and improve the efficacy of RCC treatments. Further studies are warranted to elucidate the precise mechanisms involved in EVs-mediated drug resistance and metabolic reprogramming in RCC and other cancers.

## 3 Advancements in the diagnosis of RCC via EVs

Non-invasive or minimally invasive diagnosis methods of RCC are highly desirable, and an increasing number of studies have identified cargo-containing EVs as promising biomarkers for the early identification of RCC. For example, Horie et al. (66) conducted a study to investigate the clinical relevance of sEVs’ γ-glutamyl transpeptidase (GGT) activity as a possible biomarker for RCC diagnosis and prognosis. The authors discovered that serum sEVs’ GGT expression was considerably more elevated in patients with advanced RCC and microvascular infiltration, implying that it may be used as a clinical marker for advanced clinical and pathological features in RCC patients. Furthermore, the preoperative serum sEVs’ GGT assay combined with pathological examination of resected specimens during surgery could predict the possibility of tumor invasion of microvessels, opening up new possibilities for RCC evaluation and long-term monitoring. Xp11.2 translocation renal cell carcinoma (Xp11 tRCC) is an uncommon sporadic child RCC for which early detection methods are still lacking. Kurahashi et al. (67) constructed a mouse model of Xp11 tRCC and found that miR-204-5p levels were significantly higher in urinary sEVs of the Xp11 tRCC mouse model than in controls. They thus proposed that miR-204-5p in urine sEVs might be a promising preliminary diagnostic biomarker for Xp11 tRCC. Niu et al. (68) developed a novel sEVs-associated lncRNA model based on the Bayesian spike-and-slab lasso method, prognostic levels of ccRCC patients were successfully predicted by several lncRNAs and highlighting its potential as a prognostic marker for RCC. Nevertheless, the utilization of sEVs-contained cargoes as biomarkers for RCC diagnosis and prognosis poses significant challenges compared to traditional tumor markers, imaging, and pathological diagnosis. The current limitations of sEVs extraction technology and extraction cost further complicate the clinical application of sEVs-carried cargo as tumor markers. Therefore, urgently needed are the development of techniques for large-scale and rapid extraction and purification of sEVs from human fluids, and the establishment of uniform standards for sEVs cargo analysis. Given these challenges, there is still a long way to go before EVs cargoes can be used as reliable markers for RCC. To summarize some of the promising EVs cargoes that could potentially serve as candidate RCC markers, we have provided a table (**Table 1**) instead of going into further details.

**Table 1:** Cargoes in EVs as a biomarker for RCC.

TYPE	Source	Number of cases	Cargoes	Expression	Method	Year	References
Protein	Tissue	13 pairs of ccRCC tissue	CA9; CD70; CD147	Upward	IHC, WB	2020	(69)
	Tissue	95 pairs of RCC tissue	LAIR1	Upward	IHC, WB, qPCR	2018	(46)
	Tissue	20 pairs of ccRCC tissue	AZU1	Upward	WB, LC-MS/MS,	2017	(60)
	Serum	133 patients with stage I and II ccRCC, 76 patients with stage III and IV	CD103	Upward	Flow Cytometry	2019	(35)
	Urine	6 ccRCC patients	PTRF	Upward	WB	2020	(70)
mRNA	Plasma	61 ccRCC patients	*TIMP-1*	Upward	RT-qPCR	2020	(71)
	Serum	35 RCC patients,19 normal	*MYO15A*	Upward	RT-qPCR	2022	(72)
	Urine	33 ccRCC patients,22 normal, 13 non-ccRCC	*GSTA1*; *CEBPA*; *PCBD1*	Downward	Microarray, RT-qPCR	2016	(73)
miRNA	Tissue	31 ccRCC patients (stages I and II) and 22 ccRCC patients (stages III and IV)	miR-15a	Upward	RT-qPCR	2021	(45)
	Plasma	69 ccRCC patients	miR-301a-3p	Upward	RT-qPCR	2020	(74)
	Plasma	31 RCC patients	miR-31-5p	Upward	RT-qPCR	2019	(65)
	Serum	132 RCC patients, 50 normal	miR-155	Upward	RT-qPCR	20211	(50)
	Serum	18 RCC patients, 7 normal	miR-4525	Upward	Microarray, RT-qPCR	2021	(75)
	Urine	70 ccRCC patients, 30 early‐stage prostate cancer patients, 30 early‐stage bladder cancer patients, 30 normal	miR-30c-5p	Upward	Next‐Generation Sequencing, RT-qPCR	2019	(76)
lncRNA	Plasma	32 RCC patients	lncARSR	Upward	RT-qPCR	2016	(20)
	Tissue	40 RCC patients	lncHILAR	Upward	Microarray, RT-qPCR	2021	(52)
circRNA	Tissue and serum	24 pairs of RCC tissue, 6 RCC blood samples, 6 normal blood samples	circ-PRKCI	Upward	RT-qPCR	2023	(44)
	Serum	30 RCC patients, 30 normal	circSAFB2	Upward	RT-qPCR	2022	(18)

## 4 The “Troika” of EVs in the treatment of RCC

**Figure 4:**
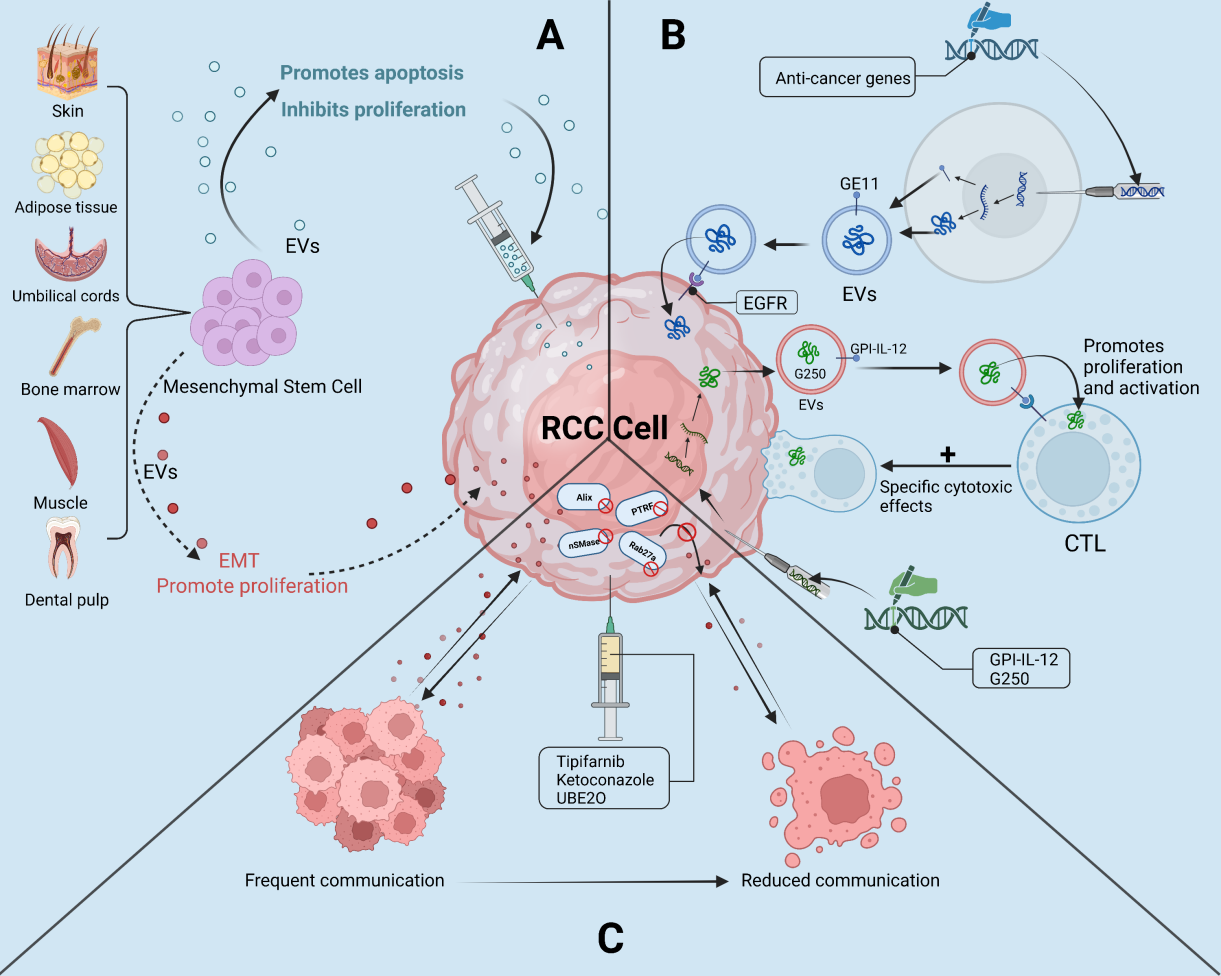
The “troika” of EVs in the treatment of RCC. **(A)**: Mesenchymal Stem Cell-derived EVs as a “double-edged sword” in the treatment of RCC; **(B)**: Potential of engineered EVs and EVs tumor vaccines in the treatment of RCC; **(C)**: By selectively reducing the secretion of tumor-derived EVs, the reduction of RCC cells with tumor microenvironment or the ability of other tumor cells to communicate with.

Recent research has demonstrated that EVs have the potential to be used as a cell-free therapy in the treatment of RCC. EVs can be engineered to deliver drugs or nucleic acids to target cells or to modulate immune responses against tumor cells. Furthermore, In preclinical investigations, EVs produced from anti-tumor cells, including mesenchymal stem cells, showed promising outcomes in battling RCC development and metastasis. Finally, targeting the secretion of tumor-derived EVs represents a promising strategy for slowing RCC development (**Figure 4**).

### 4.1 Engineered sEVs and tumor vaccines

The engineering of sEVs offers a versatile platform for tailoring the properties of sEVs to enhance their targeting specificity for tissues and cells, and to load them with therapeutic cargoes for improving disease treatments (77). Engineering sEVs has emerged as a promising approach to improve the safety and effectiveness of sEVs-based therapeutics in cancer and other diseases (78–80). Through surface modifications or the incorporation of therapeutic moieties, engineered sEVs can achieve precise delivery of therapeutics, thus enhancing their selective accumulation at the tumor site, while reducing non-specific dissemination throughout the body (81). These advancements have highlighted the enormous potential of engineered sEVs for safe, targeted, and effective drug delivery, paving the way for the development of more advanced and personalized therapies (82).

Adem et al. (83) performed experiments with engineered sEVs in ccRCC. Specifically, the authors incorporated a peptide named GE11 onto sEVs that were being secreted by 293T cells to specifically target the abnormally high levels of EGFR in ccRCC cells. Remarkably, the engineered sEVs overexpressing GE11 were shown to be preferentially absorbed by ccRCC cells rather than normal kidney cells, thus providing a specific delivery system for anticancer drugs. Although unfortunately, this article does not document more experimental results and detailed information, it is still a very interesting point about engineered sEVs that may be an excellent cell-free strategy for the therapy of many diseases, including RCC. Further research is necessary to fully elucidate the potential of this approach and determine its clinical feasibility.

In addition, it has been shown that by combining the immunomodulatory function of sEVs with their drug carrier properties, engineered sEVs can serve as potent tumor vaccines that can stimulate anti-tumor immune responses alongside chemotherapy (84). For example, Zhang et al. (85) constructed a mammalian co-expression plasmid of glycolipid-anchored-IL-12 (GPI-IL-12) and transfected GPI-IL-12 into RCC cells so that its secreted sEVs expressed GPI-IL-12 and renal cell carcinoma-associated antigen G250, this sEVs exhibited excellent antitumor effects, and its strong immunogenicity can exacerbate the cytotoxic effect on tumor cells by inducing the generation of antigen-specific cytotoxic T lymphocytes. In addition, RenCa cells secreted sEVs (RDE) can stimulate an increase in the proportion of CD8^+^ T cells by activating the FasL/Fas signaling pathway, and the cytotoxic effect of CD8^+^ T cells is enhanced after ingestion of RDE, which has specific cytotoxicity to RCC cells, this experimental result is expected to promote the development of RCC vaccine (86).

Although there are still few studies on engineered sEVs and sEVs tumor vaccines in RCC, such efforts offer great potential for advancing the field of personalized medicine for RCC treatment. By highlighting these critical aspects of research, we aim to promote further breakthroughs in engineered sEVs and sEVs tumor vaccine development for RCC.

### 4.2 Mesenchymal stem cell-derived EVs therapy

Because of the anti-inflammatory and immunomodulatory effects exhibited in preclinical and clinical research, mesenchymal stem cell-derived EVs (MSCs-EVs) have emerged as a viable strategy for cell-free therapy in a variety of disorders (87). In comparison with traditional MSCs-based therapies, the advantages of MSCs-EVs in terms of safety, stability, and preservation have provided a strong impetus for the development of MSCs-EVs-based treatments for a variety of disorders, including cancer (88), Covid-19 (89), neurological disorders (90), spinal cord injuries (91), and another kidney disease (92). These investigations have emphasized the promise of MSCs-EVs as a cell-free alternative to MSCs-based treatments, offering efficient and safer effects, and pointing to an exciting new avenue for regenerative medicine (93).

Li et al. (94) reported that intravenous administration of human umbilical cord mesenchymal stem cell sEVs (hucMSC-sEVs) in an animal model of RCC showed enhanced T cell-mediated immune responses against ccRCC via upregulation of miR-182, which inhibited VEGFA expression and delayed ccRCC progression. Similarly, Fonsato et al. (95) demonstrated that co-treatment with sEVs secreted from human liver stem cells (HLSC-sEVs) and TKI enhanced the anticancer efficacy of TKI by inhibiting the mTOR signaling pathway. Moreover, further studies revealed that HLSC-derived sEVs harbored abundant anticancer miRNAs, including miR-Let7b, miR-200b, miR-200c, and miR-223, which greatly suppressed the proliferation, invasiveness, and promoted apoptosis of RCC stem cells *in vitro*. Treatment with HLSC-sEVs was found to effectively inhibit tumor growth and angiogenesis by delivering miR-145, which downregulates EGFR and MMP1. The treatment also demonstrated the ability to attenuate tumor metastasis to the lung and significantly improve survival in a mouse model of RCC stem cell induction (96). These studies highlight the curative value of EVs produced from diverse stem cells as innovative RCC therapy techniques and offer new avenues for the development of cell-free therapies that target RCC.

However, stem cell EVs therapy is also a double-edged sword, as MSCs-EVs have also been found to have pro-tumor effects in many tumors. This may be caused by the source of the EVs cargoes or MSCs. For instance, the miR-21-5p carried by MSCs-sEVs induces the production of M2 polarization by targeting PTEN in macrophages, leading to the suppression of immune function in lung cancer (97). miR-301b-3p in MSCs-EVs has been shown to contribute to multidrug resistance in gastric cancer cells (98), and miR-193a-3p, miR-210-3p, and miR-5100 carried by bone marrow mesenchymal stem cells-derived sEVs can contribute to the progression of lung cancer by promoting EMT (99). Although the precise mechanisms underlying the dual role of MSCs-EVs remain unclear, reducing the potential risks associated with MSCs-EVs therapies in cancer will be a critical focus of future research.

Due to the potential risk of MSCs-EVs promoting cancer, which may be associated with the cargo they carry, current research aims to reduce the potential risks associated with using MSCs-EVs as a cancer treatment. One approach to achieving this is to artificially package drugs into MSCs-EVs, thereby using them as drug delivery systems (100). Studies have shown that MSCs-EVs have excellent tumor-targeting capabilities, and due to their low immunogenicity, they are not cleared by the immune cells. For example, Bagheri et al. (101) used electroporation to package doxorubicin into MSCs-sEVs and found that the drug-carrying MSCs-sEVs could effectively and safely target and kill colon cancer cells in mice. Melzer et al. (102) pretreated MSCs with paclitaxel before extracting MSCs-sEVs, allowing paclitaxel to be encapsulated into sEVs. Although the concentration of paclitaxel in MSCs-EVs was not ideal, compared with untreated MSCs-sEVs, paclitaxel-containing MSCs-EVs showed effective tumor-targeting properties and cytotoxicity in breast, lung, and ovarian cancer.

To enhance the tumor-specific targeting ability of MSCs-sEVs, it is necessary to combine MSCs-sEVs with engineered EVs technology. Using genetic engineering techniques, highly expressed factors in different tumors can be fixed on the membrane structure of MSCs-sEVs, and different targeting molecules can be selected according to different types of cancer (103). By artificially packaging anticancer drugs into MSCs-sEVs, it is theoretically possible to achieve a safe and effective MSCs-EVs cell-free anti-tumor therapy. However, it is still necessary to further understand the complex interactions between tumor cells and MSCs-EVs, which is critical for developing safer and more effective treatment strategies.

### 4.3 Reduced secretion of tumor-derived EVs

Tsuruda et al. (104) observed RAB27B overexpression in sunitinib-resistant RCC cells, suggesting its role in conferring drug resistance. Additionally, RAB27B is essential in the biogenesis of sEVs. Although the knockdown of RAB27B reduced the viability of RCC cells, it had little effect on sEVs secretion. However, this observation raises significant implications for a potential therapeutic target in RCC through two distinctive pathways. First, as mentioned earlier, the knockdown of RAB27B directly inhibits the aggressive and proliferative properties of RCC cells. Secondly, previous studies have demonstrated that tumor-secreted sEVs significantly contribute to the progression of RCC. By suppressing the production of sEVs through the inhibition of RAB27B or other sEVs-related proteins, it is possible to weaken or disrupt the communication between RCC cells and neighboring cells in the TAM. Experiments performed by other researchers have quickly validated this perspective.

Greenberg et al. (105) have conducted several studies on drug combination therapy for sEVs-associated RCC, and their findings reveal that the combination of ketoconazole and sunitinib increases the anticancer effect of sunitinib. Ketoconazole selectively reduces sEVs formation and transport in RCC cells by targeting the ERK signaling pathway to inhibit proteins associated with sEVs production and secretion in RCC cells, thereby inhibiting the proliferation of RCC cells and enhancing the anticancer efficacy of sunitinib. Another study found that sunitinib-resistant RCC cell-derived sEVs had greater amounts of PD-L1, which promoted the death of T cells by PD-1 and PD-L1 interactions and led to immunological tolerance. Similarly, tipifarnib suppressed sEVs formation and release via ESCRT-dependent along with non-dependent routes, while also exhibiting synergistic interactions with sunitinib to combat drug resistance and cause death in resistant cells (106). Taken together, these studies suggest that ketoconazole or tipifenib has the potential to act as an adjuvant to reduce the secretion of tumor-derived sEVs during the treatment of RCC in combination with sunitinib. Furthermore, the increased expression of Polymerase I and Transcript Release Factor (PTRF) associated with ccRCC cells and the sEVs they secrete, so the tumors secreted more sEVs compared to normal tissue, and Cen et al. (107) have demonstrated that Ubiquitin-conjugating enzyme E2O (UBE2O) can reduce the number of sEVs secreted by ccRCC cells by ubiquitinating PTRF, thus reducing caveolae formation which is involved in sEVs biogenesis. By suppressing the release of EVs by cancer cells, downstream signaling pathways that promote RCC growth, metastasis, drug resistance, and immune evasion may be disrupted. Therefore, this approach holds great potential for developing effective therapies for RCC patients.

## 5 Summary

EVs are found in nearly all body fluids and play a significant part in numerous biological functions in humans. This study gives a review of the developments achieved in the knowledge of EVs in the etiology and therapy of RCC. Tumor cells can exchange substances with the microenvironment and neighboring cells through their secreted EVs. These sEVs can significantly affect the microenvironment, suppress the immune system while promoting tumor growth and metastasis, and also confer drug resistance to neighboring tumor cells. Overall, the study of EVs in RCC presents an exciting avenue of research with significant potential for improving the diagnosis and therapy of this disease. More research is required to completely understand the molecular pathways behind the participation of EVs in RCC and to maximize their diagnostic and therapeutic applications. Nonetheless, the rapid progress in this field suggests that EV-based approaches may soon alter the way we treat RCC.

Nonetheless, there remain considerable challenges that must be surmounted before these promising findings can be translated into clinical practice. Firstly, researchers must optimize existing methods for the isolation and preparation of EVs and establish uniform standards to ensure the reproducibility of results across different laboratories. Second, extensive testing is required to determine the security and effectiveness of EV-based therapeutics, as well as to assess any potential synergistic benefits with existing treatment modalities. Importantly, a thorough understanding of the molecular mechanisms underpinning EVs’ function in RCC is also required. Only with a comprehensive understanding of these mechanisms can EV-based strategies be optimized for personalized and effective therapies.

Looking toward the future, the field of EVs in RCC research is expected to grow rapidly, propelled by enhancements in EVs biology, nucleic acid sequencing, and gene editing technologies. It is hoped that these advances will facilitate the development of personalized treatments that take into consideration the distinct molecular and genetic characteristics of individual patients. Overall, the study of EVs in RCC represents an intriguing area of research that holds great promise for better understanding and treating this deadly disease.

## Author contributions

YL finished collecting and analyzing relevant material, writing the first draft of the paper, and designing diagrams. MZ and JZ assisted in the initial writing, graph design, and data gathering. The investigation and categorization of the literature, the creation of the diagrams, and the review of the semantics were all done by XL and LW The graphs were designed by YM and the papers were collected by XH, RG wrote and reviewed the articles in the table to make grammatical corrections. ZC chose the topic, directed the drafting of the paper, gave final approval of the published version, chose the journal to which the paper would be submitted, and agreed to be accountable for all elements of the work. All authors contributed to the article and approved the submitted version.

## Glossary

**Table:**

Alix	Alg-2 Interacting Protein X
ApoC1	Apolipoprotein C1
AXL	Anexelekto
AZU1	Azurocidin 1
BMSCs-Exos	Bone Marrow Stromal Cells-Derived Exosomes
BTG2	B-Cell Translocation Gene 2
CA9	Carbonic Anhydrase 9
CAFs	Cancer-Associated Fibroblasts
CCND1	Cyclin D1
ccRCC	Clear Cell Renal Cell Carcinomas
CD	Clusters Of Differentiation
CircRNA	Circular RNA
c-MET	Cellular-Mesenchymal Epithelial Transition Factor
CSF-1	Colony Stimulating Factor 1
CTLs	Cytotoxic T Lymphocytes
CXCR4	C-X-C Motif Chemokine Receptor 4
DC	Dentritic Cell
DMEM	Dulbecco’s Modification Of Eagle’s Medium
EGFR	Epidermal Growth Factor Receptor
EMT	Epithelia-Mesenchymal Transition
ERK	Extracellular Regulated Protein Kinases
ESCRT	Endosomal Sorting Complex Required For Transport
ETS1	ETS Proto−Oncogene 1, Transcription Factor
EVs	Extracellular Vesicles
FGF2	Fibroblast Growth Factor2
FOXO3	Forkhead Box O3
GGT	Γ-Glutamyl Transpeptidase
GPI-IL-12	Glycolipid-Anchored-IL-12
hepaCAM	Hepatocyte Cell Adhesion Molecule
HIF1A	Hypoxia-Inducible Factor 1A
HIF1α	Hypoxia-Inducible Factor-1α
HLA-G	Human Leukocyte Antigen-G
HLSC-Exos	Human Liver Stem Cell-Derived Exosomes
HSP70	Heat Shock Protein 70
hucMSC-Exos	Human Umbilical Cord Mesenchymal Stem Cell Exosomes
HuR	Human Antigen R
IGF1R	Insulin Like Growth Factor 1 Receptor
IL	Interleukin
JAK1	Janus Kinase 1
LAIR1	Leukocyte-Associated Immunoglobulin-Like Receptor 1
LncRNA	Long Non-Coding RNA
MALAT1	Metastasis-Associated Lung Adenocarcinoma Transcript 1
MDSCs	Myeloid-Derived Suppressor Cells
miRNA	Micro RNA
MLH1	Mutl Homolog 1
MMP1	Matrix Metallopeptidase 1
MMP3	Matrix Metallopeptidase 3
MSCs	Mesenchymal Stem Cells
MSCs-EVs	Mesenchymal Stem Cell-Derived Evs
MSCs-Exos	Mesenchymal Stem Cell-Derived Exosomes
mTOR	Mammalian Target Of Rapamycin
MVBs	Multivesicular Bodies
MVs	Microvesicles
NF-κB	Nuclear Factor Kappa-B
NK cells	Natural Killer Cell
PBS	Phosphate Buffer Solution
PD-L1	Programmed Cell Death-Ligand 1
PKB/Akt	Protein Kinase B
PTEN	Phosphatase And Tensin Homolog
PTRF	Polymerase I And Transcript Release Factor
RCC	Renal Cell Carcinoma
RDE	Renca Cell-Derived Exosomes
RNF43	Ring Finger Protein 43
STAT3	Signal Transducer And Activator Of Transcription 3
TAM	Tumor-Associated Macrophage
TFCP2L1	Transcription Factor Cp2 Like 1
TGF-β	Transforming Growth Factor-Beta
TG-RS	Time-Gated Raman Spectroscopy
TIMP2	Tissue Inhibitors Of Metalloproteinase 2
TKI	Tyrosine Kinase Inhibitor
TLR2	Toll-Like Receptor 2
TME	Tumor Microenvironment
TSG101	Tumour Susceptibility Gene 101
UBE2O	Ubiquitin-Conjugating Enzyme E2O
VEGF	Vascular Endothelial Growth Factor
Xp11 tRCC	Xp11.2 Translocation Renal Cell Carcinoma
